# High numerical aperture imaging allows chirality measurement in individual collagen fibrils using polarization second harmonic generation microscopy

**DOI:** 10.1515/nanoph-2023-0177

**Published:** 2023-04-14

**Authors:** MacAulay Harvey, Richard Cisek, Mehdi Alizadeh, Virginijus Barzda, Laurent Kreplak, Danielle Tokarz

**Affiliations:** Department of Chemistry, Saint Mary’s University, 923 Robie Street, Halifax, NS, B3H 3C3 Canada; Department of Chemical and Physical Sciences, University of Toronto Mississauga, Mississauga, ON, L5L 1C6, Canada; Department of Physics, University of Toronto, 60 St. George St, Toronto, ON, M5S 1A7, Canada; Laser Research Center, Faculty of Physics, Vilnius University, Sauletekio Av. 9, LT-10222 Vilnius, Lithuania; Department of Physics and Atmospheric Science and School of Biomedical Engineering, Dalhousie University, Halifax, NS, B3H 4J5, Canada

**Keywords:** atomic force microscopy, nano-biophotonics, nanofibers, nanoscale structures, nonlinear optical microscopy, polarization-resolved imaging

## Abstract

Second harmonic generation (SHG) microscopy is a commonly used technique to study the organization of collagen within tissues. However, individual collagen fibrils, which have diameters much smaller than the resolution of most optical systems, have not been extensively investigated. Here we probe the structure of individual collagen fibrils using polarization-resolved SHG (PSHG) microscopy and atomic force microscopy. We find that longitudinally polarized light occurring at the edge of a focal volume of a high numerical aperture microscope objective illuminated with linearly polarized light creates a measurable variation in PSHG signal along the axis orthogonal to an individual collagen fibril. By comparing numerical simulations to experimental data, we are able to estimate parameters related to the structure and chirality of the collagen fibril without tilting the sample out of the image plane, or cutting tissue at different angles, enabling chirality measurements on individual nanostructures to be performed in standard PSHG microscopes. The results presented here are expected to lead to a better understanding of PSHG results from both collagen fibrils and collagenous tissues. Further, the technique presented can be applied to other chiral nanoscale structures such as microtubules, nanowires, and nanoribbons.

## 1 Introduction

Collagen fibrils are nanoscale rope-like structures and a major structural component of tissues such as tendons, cornea, and skin. The structure and function of these fibrils have been investigated using a number of techniques such as atomic force microscopy (AFM), X-ray diffraction, and scanning electron microscopy among others [1–5]. Collagen fibrils are composed of a right-handed supertwisted arrangement of collagen microfibrils [4, 6], which are themselves comprised of a left-handed arrangement of right-handed triple helical tropocollagen molecules [4, 7, 8]. This twisted structure has led to the characterization of collagen fibrils as nanoscale biological ropes due to their similarities in both structure and function [9]. Due to the high molecular hyperpolarizability of tropocollagen arrangements in collagen types I–III and owing to their non-central symmetry on all levels of structural hierarchy, collagen fibrils can double the frequency of an incident beam of light, in a process known as second harmonic generation (SHG) [10, 11]. It has been shown that polarization-dependent SHG (PSHG) signal is sensitive to the organization of tropocollagen molecules within a fibril, the chirality of the collagen molecule as well as the overall chirality of the fibril [12–15]. PSHG microscopy has been used previously to study the arrangement of collagen fibrils within collagenous tissues such as tendon, skin, cornea, cartilage, and bone [14, 16], [17], [18], [19], [20], [21], [22], and has been suggested to be a potential tool for differentiating diseased from normal tissue including esophagus, ovary, thyroid, pancreas, and lung among others [23–28].

However, since individual collagen fibrils typically have diameters within the range of 20–500 nm [29], it is difficult to probe their internal structure using PSHG due to the diffraction limited resolution of optical microscopy techniques. PSHG has been used to understand how certain factors such as aging and fibril rupture result in a relative change in the structure of isolated collagen fibrils [30, 31]. Comparison of PSHG data to theoretical models of fibril PSHG response has enabled parameters such as the angle of the tropocollagen molecule with respect to the fibril axis, and pitch angle of the collagen triple helix to be estimated however, these techniques have so far only been applied to fibrils in tissues [12, 32, 33], where there can be multiple fibrils at different orientations present in a single focal volume potentially leading to experimental uncertainty.

Here we demonstrate a new technique where the transverse-to-fiber PSHG structural parameter profile, obtained at high numerical aperture (NA), is used to investigate the ultrastructure of individual collagen fibrils. By fitting numerical simulations to experimental data, we are able to estimate the values of parameters relating to the structure and chirality of the fibril, and unlike other known PSHG techniques, we are able to obtain values for these parameters without tilting the fibril away from the optical plane, or cutting the tissue at different angles [14, 16, 34, 35]. Our results indicate that PSHG utilizing the edge effect in high NA focal volumes is a useful tool for the investigation of the internal structure of individual collagen fibrils. Importantly, the technique described here has potential to be used to analyze the structure of other biological chiral nanoscale SHG emitters including myofilaments and microtubules as well as synthetic chiral nanostructures such as nanowires.

## 2 Materials and methods

### Collagen fibril isolation

Collagen fibrils are extracted from ∼2 mm thick sections cut from dissected adult bovine and ovine lateral digital extensor tendons sourced from a local abattoir and kept at −80 °C before use. The tendon section is hydrated with 1 mL of reverse osmosis water and fibrils are extracted into the surrounding liquid by scraping the tissue with tweezers for approximately 5 min as described in [3]. Then 2 µL of the solution containing the fibrils is subsequently deposited onto a glass coverslip (16,004-314, VWR) using a pipette. The fibrils are allowed to sink and adhere to the substrate for ∼45 min, and then the substrate is washed with reverse osmosis water for approximately 1 min and dried using a stream of nitrogen gas for approximately 5 min. The fibrils on glass are left to air dry overnight and are imaged within a week of isolation. No coverslip is placed on top of the fibrils before imaging.

### Polarization-in, polarization-out SHG imaging

**Figure 1:**
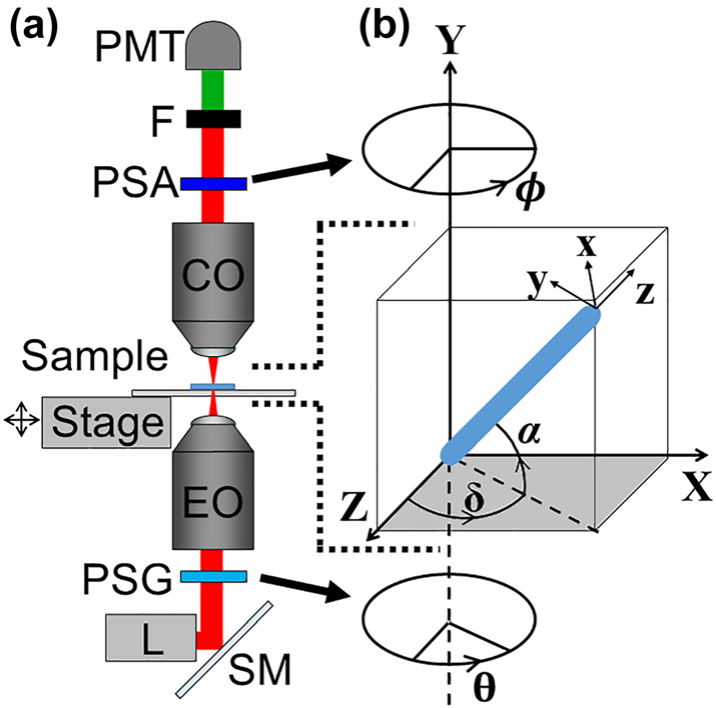
Schematic diagram showing the SHG microscope (a) and the coordinate systems used (b), where a collagen fibril at an arbitrary orientation is represented as a thick blue cylinder. The following abbreviations are used in (a): L-laser, SM-scanning mirrors, PSG-polarization state generator, EO-excitation objective, CO-collection objective, PSA-polarization state analyzer, F-optical filter, and PMT-photomultiplier tube detector. The focal plane is the *Z*–*X* plane and is shown in grey in (b).

Polarization-in, polarization-out SHG (PIPO-SHG) images were obtained using a custom-built laser scanning microscope, previously described in [36] and is schematically shown in Figure 1(a). Briefly, the laser beam was raster scanned across the sample using a pair of galvanometric scan mirrors (ScannerMAX, Pangolin Laser Systems, Inc.) with a pixel dwell time of 6 µs, to create an image with 100 × 100 pixels and size 18 × 18 µm. The beam was focused onto the sample using an air immersion objective lens with NA of 0.8, or 0.5 (Plan-Apochromat 20×, or Plan-Neofluar 20×, Carl Zeiss AG). The sample was placed such that the laser beam would pass through the coverslip before encountering the fibrils. SHG signal was collected in transmission geometry using a custom objective lens with an NA of 0.85 (Omex Technologies, Inc.). The ultrafast laser (FemtoLux 3, EKSPLA) had a wavelength of 1030 nm, repetition rate of 5 MHz, and pulse duration of ∼290 fs. The SHG signal was separated from the laser light using an interference filter centered at 515 nm with a 10 nm bandwidth (65–153, Edmund Optics Inc.). SHG signal was measured using a single-photon-counting photomultiplier detector (H10682-210, Hamamatsu Photonics K.K.) and obtained using a data acquisition card (PCIe-6363, NI).

The polarization state generator (PSG) consisted of a stationary linear polarizer (LPVIS100, Thorlabs, Inc.) followed by a flat half-wave plate (WPMP2-22-V1030, Karl Lambrecht Corp.) on a motorized rotation stage [see Figure 1(a)]. The polarization state analyzer (PSA) consisted of a polarizing filter (analyzer, LPVISA 100, Thorlabs, Inc.) on a motorized rotation stage. A PIPO-SHG image stack is produced by obtaining SHG images of the sample at all combinations of eight laser polarization angles and eight analyzer angles at 22.5° increments for a total of 64 images. At the end, a final image is obtained at the same half-wave plate and analyzer angles as the first image as a control for photobleaching or movement of the sample. The PIPO-SHG data is fit using a trust region algorithm in MATLAB (The Mathworks, Inc.) using Equation (1) which describes the SHG intensity (*I*_SHG_) as a function of laser linear polarization angle (*θ*′) and analyzer angle (*φ*′) from a sample under the assumption that the fibril is cylindrically symmetric and letting $\chi_{\mathrm{xxz}}^{\left(2\right)}$ = $\chi_{\mathrm{zxx}}^{\left(2\right)}$ as is typical for rod like structures [14, 37] and neglecting potential contributions from local field effects such as from charge on the fibril or the coverslip [38]:

(1) $$\begin{array}{ll} I_{\text{SHG}} & =A|\rho \cos\phi^{\prime }\cos^{2}\theta^{\prime }+\sin\varphi^{\prime }\sin2\theta^{\prime }\\ & \;+\cos\phi^{\prime }\sin^{2}\theta^{\prime }+2\kappa \cos\phi^{\prime }\sin\theta^{\prime }{|}^{2}+F \end{array}$$

Here *A* is a constant of proportionality between the SHG electric field and measured intensity, *F* is the noise floor, and the structural parameters *ρ* and *κ* are defined as:

(2a) $$\rho =\frac{\chi_{\mathrm{ZZZ}}^{\left(2\right)}}{\chi_{\mathrm{ZXX}}^{\left(2\right)}}=\rho_{f}\cos^{2}\alpha +3\sin^{2}\alpha \;\;\;\;\;\;\rho_{f}=\frac{\chi_{\mathrm{zzz}}^{\left(2\right)}}{\chi_{\mathrm{zxx}}^{\left(2\right)}}$$

(2b) $$\kappa =\frac{\chi_{\mathrm{XY Z}}^{\left(2\right)}}{\chi_{\mathrm{ZXX}}^{\left(2\right)}}=\kappa_{f}\sin\alpha \;\;\;\;\;\;\kappa_{f}=\frac{\chi_{\mathrm{xyz}}^{\left(2\right)}}{\chi_{\mathrm{zxx}}^{\left(2\right)}}$$

where *α* is the tilt angle of the fibril from the focal plane [*Z*–*X*, see Figure 1(b)], and $\chi^{\left(2\right)}$ is the second order optical electric susceptibility tensor of the collagen fibril which relates the induced polarization in a material to the applied electric fields. The laser is directed along *Y* in the laboratory coordinate system, *XYZ*, while the fibril is oriented along *z* in the fibril frame coordinate system, *xyz*, as shown in Figure 1(b). The angles *θ* and *ϕ* are defined such that *θ*′ = *θ* − *δ* and *ϕ*′ = *ϕ* − *δ* where *δ* is the angle from the *Z* axis to the projection of the fibril onto the image plane. Parameters *ρ*, *κ*, *ρ*_*f*_, and *κ*_*f*_ are ratios of $\chi^{\left(2\right)}$ elements used to quantify the structure of the collagen fibril where *ρ* and *κ* are measured in the laboratory frame of reference, and *ρ*_*f*_ and *κ*_*f*_ are the corresponding values in the fibril frame of reference [14, 16]. Only pixels which can be fit using Equation (1) with goodness of fit parameter *R* ^2^ ≥ 0.8 are considered for further analysis.

### Extraction of *ρ* profile from PIPO-SHG images

Following PIPO-SHG imaging and fitting, the profiles of *ρ* values across each fibril are extracted along 20 different lines perpendicular to the fibril axis. These 20 profiles are then manually aligned, averaged, and their standard deviation is reported as the uncertainty to produce a profile of the *ρ* values across the fibril.

### Atomic force microscopy imaging

**Figure 2:**
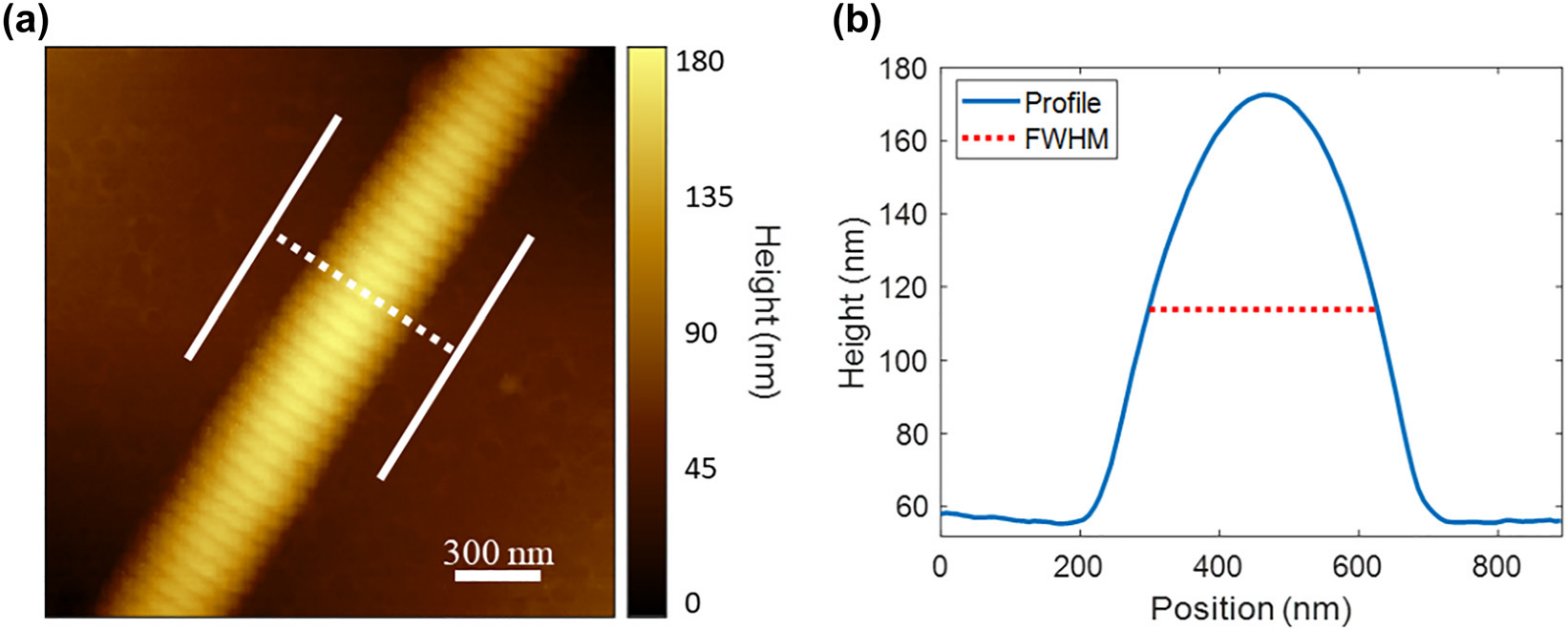
AFM data for a typical fibril. An AFM height image (a) and extracted profile (b) for one of the fibrils imaged using PIPO-SHG. The white dotted line in (a) represents the line profile in (b) and the two solid white lines represent the 128 pixel long region which was averaged over.

The diameter of a single collagen fibril is much smaller than the resolution of our optical system, therefore AFM imaging is used to accurately measure the diameter of fibrils used in this study. AFM imaging was performed using an Agilent 5500 system operating in acoustic mode. Cantilevers had a natural frequency around 300 kHz (TESPA-V2, Bruker Corp.), and topographical images were acquired at a scanning frequency of 1 Hz and a pixel size of 7.8 nm [Figure 2(a)]. All images were analyzed using Gwyddion [39]. Images were flattened and an average profile over 128 pixels was extracted across a straight region of the fibril and used to estimate fibril width via the full width at half-maximum (FWHM) fibril height [Figure 2(b)]. To estimate uncertainty, five profiles were extracted from different points on a fibril using 20 pixels of averaging each. The standard deviation in diameter measured from these five profiles is reported as the uncertainty.

### Numerical simulations

A previously described open source program was utilized to carry out numerical simulations [40]. Briefly, this program calculates the electric field within the volume of a microscope objective focal volume of a given NA, assuming a perfectly collimated and linearly polarized Gaussian (TEM_00_) laser beam with no longitudinally polarized component. The program then simulates the interaction of the resulting electric field with a given spatial distribution of complex-valued second order electric susceptibilities in a laser focal volume, and reports a simulated value of the detected SHG intensity. In all simulations values of $\chi_{\text{xxz}}^{\left(2\right)}$ = $\chi_{\text{yyz}}^{\left(2\right)}$ = $\chi_{\text{zxx}}^{\left(2\right)}$ = $\chi_{\text{zyy}}^{\left(2\right)}$ = 1 in accordance with a cylindrically symmetric structure, while the values of $\chi_{\text{zzz}}^{\left(2\right)}$ and $\chi_{\text{xyz}}^{\left(2\right)}$ are varied to set the desired values of *ρ*_*f*_ and *κ*_*f*_. This program utilizes chirp Z-transforms to calculate the focal field and far field intensity distributions which allows for faster calculation speed compared to some numerical integration methods [41]. All simulations are carried out using a grid of 128 × 128 points in *Z*–*X* and 64 points in *Y*, with each voxel having a size of 7.8 nm in *Z*–*X* and 37.5 nm in *Y*. NA values of 0.8 for excitation and 0.85 for collection (as in the PIPO-SHG microscope described above) are used. The collagen fibrils were assumed to be cylinders with lengths much greater than the waist size of the laser focus centered on the focal plane and oriented along *Z* in the focal plane (*α* = 0). A refractive index (*n*) of 1 was assumed for all points within the focal volume, and the effect of reflections from the fibril and coverslip interfaces was neglected.

This program is run for all 64 combinations of eight laser polarization angles and eight analyzer angles to produce a simulated set of PIPO-SHG data for a single pixel with the fibril at a given position within the focal volume. This data is then fit with the same parameters as the experimental data using Equation (1).

## 3 Results

### PIPO-SHG of individual collagen fibrils

**Figure 3:**
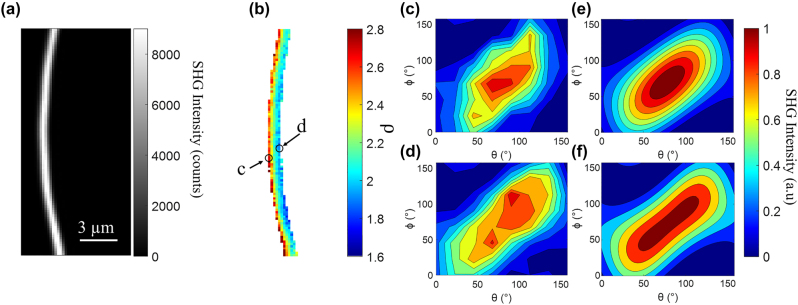
PIPO-SHG results for a typical collagen fibril. An SHG intensity image of a collagen fibril produced by summing the first 64 PIPO-SHG images (a), and a color-coded image showing the fitted values of *ρ* at each pixel (b), which also indicates two typical pixels of interest on opposing sides of the fibril (c and d). Panels (c) and (e) and (d) and (f) contain data and fits, respectively, for the corresponding pixels, visualized as contour plots of normalized SHG intensity versus laser polarization and analyzer angles. Note that the fibril shown here appears much larger than that shown in Figure 2 as a result of the low spatial resolution of the SHG microscope compared to AFM.

PIPO-SHG measurements were performed on dry collagen fibrils from tendon on glass coverslips using a 0.8 NA excitation objective to obtain a resolution of approximately 0.6 µm laterally and 3 µm axially. The PIPO-SHG data was fitted to extract values of *ρ* at each pixel as described above, and a typical result is shown in Figure 3. Laser powers in the range of 2–4 mW at the sample were used to produce sufficient SHG signal for PIPO-SHG imaging, and a typical SHG intensity image, obtained by a sum of 64 PSHG images, is shown in Figure 3(a). A sample of 5 dry fibrils was investigated by PSHG and data was fit as described above to obtain values of *ρ* and *κ*. The mean *R* ^2^ value of the fits for pixels considered here was 0.94 ± 0.04 (mean ± standard deviation). The mean value of *ρ* obtained by fitting a Gaussian function to a histogram of *ρ* values from all five fibrils combined is 2.01 ± 0.01. This value is in line with the *ρ* values we measure for dried collagen fibrils (see Figure S1 in supplementary material for a comparison of dried and rehydrated fibrils and comparison to previous measurements).

In typical fibrils a gradient in *ρ* values can be observed transverse to the axis of the fibril, as shown for the fibril in Figure 3(b), where its *ρ* values range from ∼3 to 1.5 going left to right. The contour plots presented in Figure 3(c), (d) show the raw PIPO-SHG measurement data for 2 typical individual pixels on either side of the fibril, consisting of normalized SHG intensities via colors (blue indicating 0 to red indicating 1) at different combinations of *θ* and *ϕ* angles. In this range of *ρ*, the PIPO-SHG data is very sensitive enabling differentiation by visualization if needed. These contour plots show significant differences in data obtained for the left [Figure 3(c)] versus the right side [Figure 3(d)] of the fibril, where the latter appears more elongated and contains two peaks as compared to the former with one. Figure 3(e) and (f) show the results of fitting the data shown in (c) and (d) using Equation (1). Note that the measured value of *κ* is typically distributed around zero in line with Equation (2b) and will not be considered here.

It is interesting to note here that all five fibrils imaged here, as well as nearly all fibrils we have imaged previously show this gradient in the same direction, going high to low from left to right for vertically oriented fibrils, and from top to bottom for horizontally oriented fibrils.

**Figure 4:**
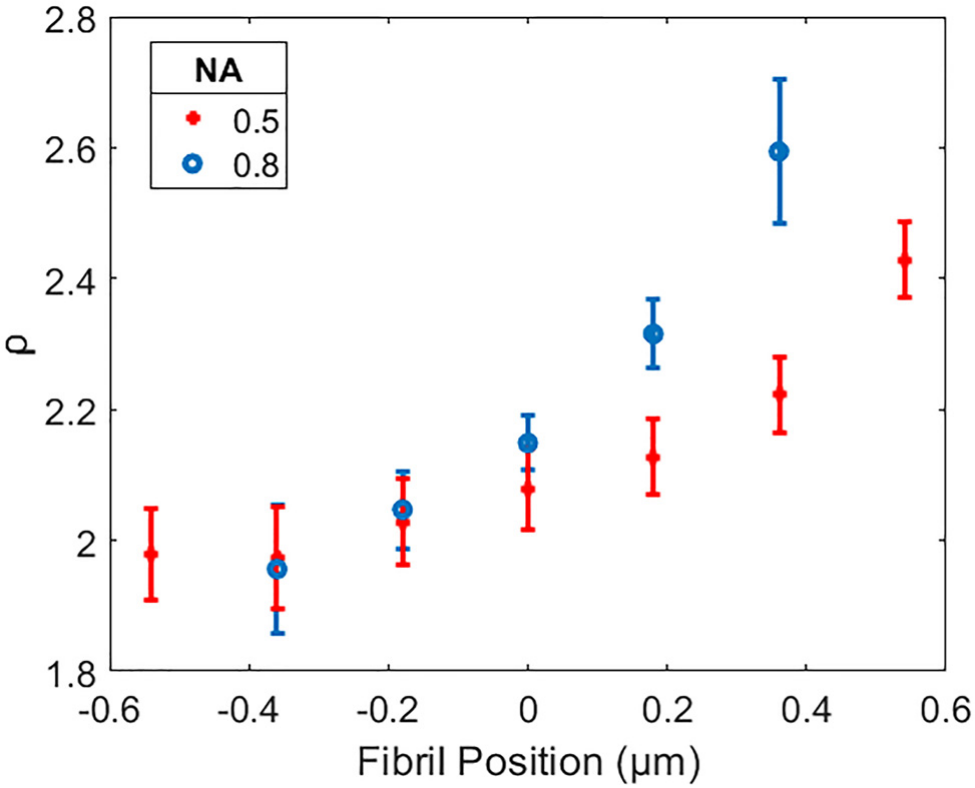
Profiles of *ρ* extracted transverse to the fibril axis from PIPO-SHG data using a 0.5 NA (red crosses) and 0.8 NA (blue dots) excitation objectives. The fibril is oriented on the focal plane along the *Z*-axis (*α* = *δ* = 0, *Y* = 0), and the graph *x*-axis indicates the fibril position from the focal plane middle (*x* = 0) along the laboratory *X*-axis [see Figure 1(b)].

This gradient is unlikely to be the result of birefringence because the effect would be uniform across the fibril. Therefore to investigate the origin of this transverse *ρ* gradient, PIPO-SHG images were obtained for a fibril using two different excitation objectives with NA of 0.5 and 0.8. Profiles of the *ρ* values across the fibril were extracted using the method outlined above, and it was found that the higher NA objective produced a much steeper gradient than the lower NA (Figure 4). As will be shown in the following section, this observation shows that the gradient likely is a result of high NA focusing of the excitation laser beam.

### Focal field polarization for a high NA objective

**Figure 5:**
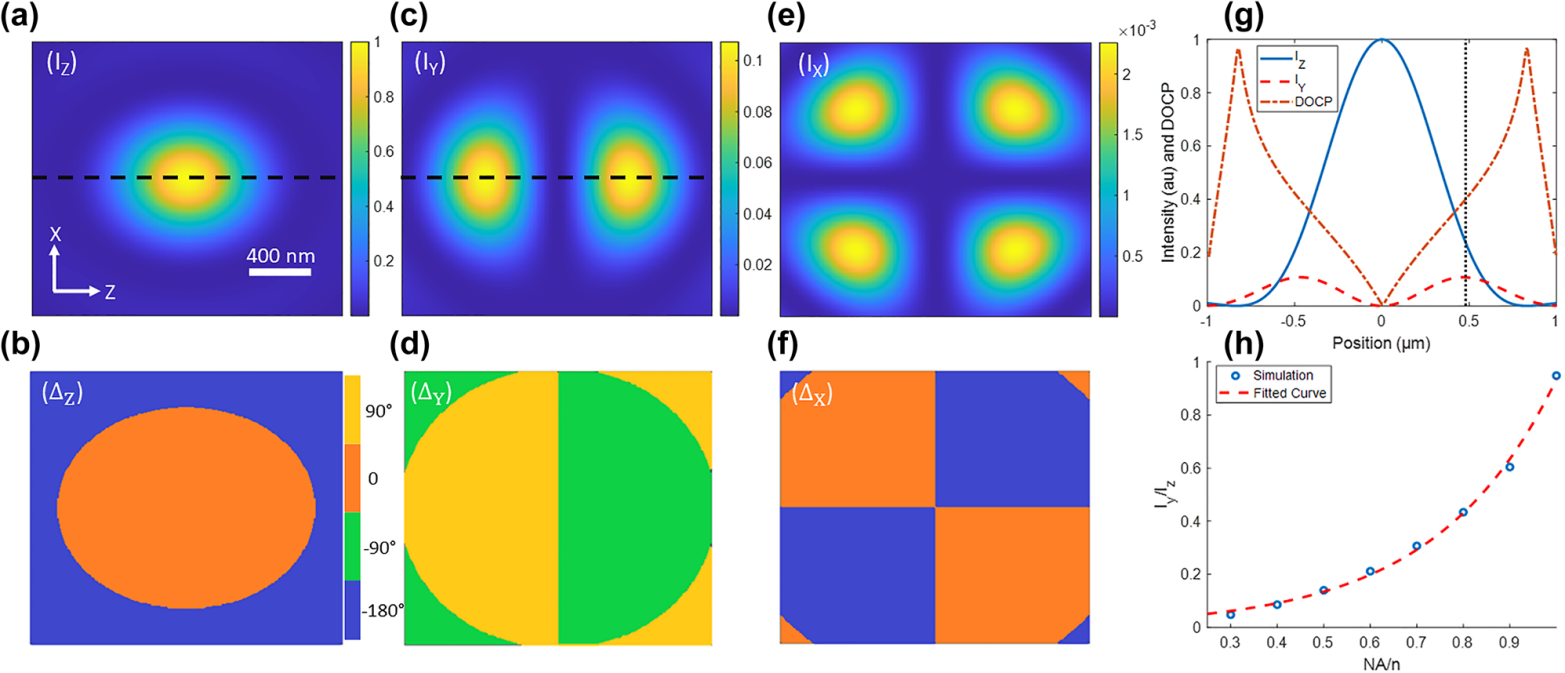
Simulations of focal plane electric field. Intensity (*I*) and phase (Δ) of the *Z* (a) and (b), *Y* (c) and (d) and *X* (e) and (f) components of the focal field for a 0.8 NA air immersion objective. The scale bar and axes in (a) apply to all six images (a)–(f), the color bar in (b) applies to (d) and (f), and (a) is normalized by its maximum intensity whereas (c) and (e) are normalized to (a). The plot in (g) shows the profiles indicated by the dotted black lines in (a) and (c), and the degree of circular polarization (DOCP) along these profiles. The plot in (h) shows the relative intensity, as a function of NA/*n* where *n* is the index of refraction, of the *Y* component of polarization as a fraction of the *Z*-polarized intensity at the quarter maximum point in *Z* intensity (half-maximum in *Z* electric field) as shown by the dotted black line in (g); open symbols are the simulation result and the broken line is an exponential fit through the data in (h).

To investigate the cause of the gradient in measured *ρ* values across collagen fibrils, numerical simulations were performed where the laser electric field has an initial polarization along *Z* (i.e. no *X* or *Y* component) and is focused by a specified NA. As has been found through previous modeling [42], the laser electric field intensity at the focal plane, modelled as the waist of the focal volume, maintains a significant polarization along *Z* [Figure 5(a)], which has a constant phase through the focal region [Figure 5(b)]. However, a longitudinal component of polarization is induced in the form of two lobes on either side of the *X* axis [Figure 5(c)], with these lobes having opposite phase from each other and are each 90° out of phase with the *Z* polarized component [compare Figure 5(b) and (d)], creating a circularly polarized light component. There is also a second transverse component polarized along *X* which takes the form of four lobes, one in each quadrant of the focal plane [Figure 5(e)], each of which have either the same or the opposite phase as the *Z* component [compare Figure 5(b) and (f)]. These additional polarization components occur as a result of the high focusing angle near the edge of a focal volume and are therefore dependent on the NA/*n* (where *n* is the refractive index of the immersion medium) [42].

The *X*-polarized component is found to have an intensity which never exceeds ∼1.5 % of the maximum *Z*-polarized intensity, and its maxima are located far from the center of the focal plane. We therefore determine that contributions from the *X*-polarized component of the focal field are unlikely to have a significant impact on PIPO-SHG measurements of collagen fibrils.

At NA 0.8 the *Y*-polarized component is found to have a much higher intensity than the *X*-polarized component. The *Y*-polarized lobes have a maximum intensity of approximately 10 % of the maximum of the *Z*-polarized component. Since fibrils typically give signal across five 180 nm pixels, therefore, the fitted PSHG pixels at the transverse edge of fibrils represent the situation where the fibril center experiences a degree of circular polarization of ∼30 % [Figure 5(g)].

To quantify the amount of longitudinally polarized light within the focal plane we compare the *Y*-polarized intensity to the *Z*-polarized intensity at a point in the focal plane where the *Z*-intensity is at one quarter maximum (*Z* electric field is at half maximum). This point, shown by the black dotted line in Figure 5(g), is found to coincide with the maximum of *Y*-polarized intensity. We find that the fraction of *Y*-polarized intensity $\frac{I_{Y}}{I_{Z}}$ increases as NA/*n* increases and that the equation $\frac{I_{Y}}{I_{Z}}\approx 0.019\exp\left(3.87NA/n\right)$ provides a good (*R* ^2^ = 0.997) approximation [Figure 5(h)].

At moderately high NAs of 0.8 and above in air the large *Y*-polarized component of the focal electric field $\left(\frac{I_{Y}}{I_{Z}}\geq 40\;\%\right)$ has potentially interesting implications for SHG microscopy. This is because for a cylindrical sample such as a collagen fibril with ∼200 nm diameter, it is possible for the sample to be off centered as compared to the focal spot, where the edge of the focal spot still illuminates the sample sufficiently to produce SHG. As a result, pixels along the edges of a collagen fibril obtained using a laser scanning microscope where the pixel size is significantly smaller than the focal spot size will have measured SHG intensity with a significant longitudinal component of the incoming polarization compared to the pixels that imaged the fibril at the center of the focal volume. Additionally, pixels on one side of the fibril will be imaged with a longitudinally polarized component of the opposite phase as compared to the other side. This longitudinal electric field component can then interact with the $\chi_{\mathrm{XY Z}}^{\left(2\right)}$ and $\chi_{\mathrm{ZY Y}}^{\left(2\right)}$ components of the second order electric susceptibility of a sample lying on the image plane, resulting in additional contributions to the measured SHG intensity which are not taken into account in Equation (1). Due to the non-uniformity of the focal electric field, each pixel across a PIPO-SHG image of a collagen fibril will experience a different longitudinal polarization, making it difficult to correct Equation (1) to take this into account.

### Numerical simulations of SHG from collagen fibrils

**Figure 6:**
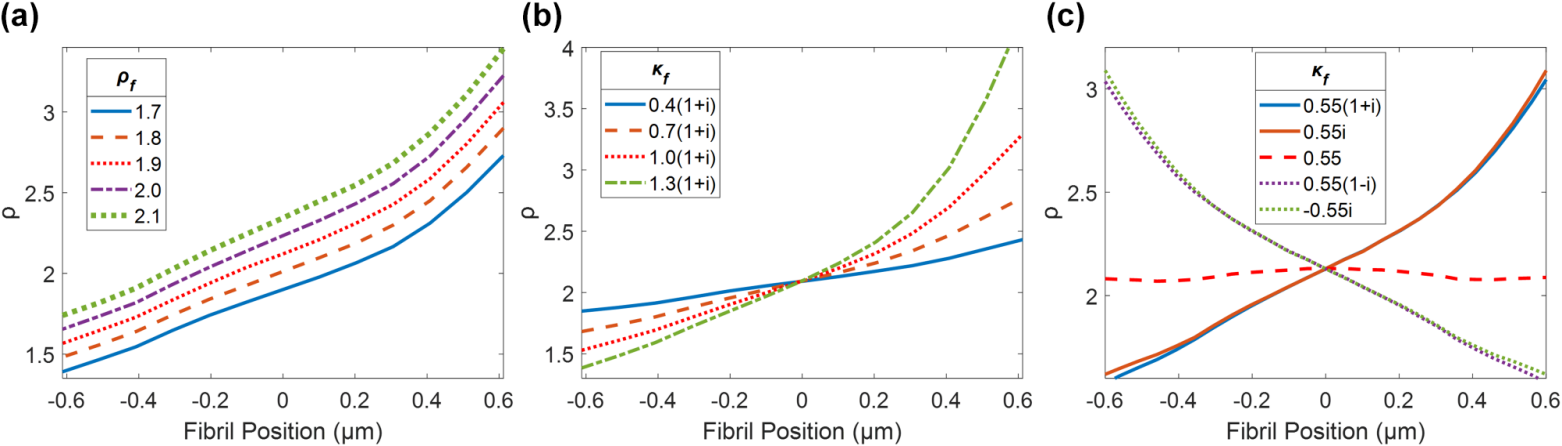
Effects of changing *ρ*_*f*_ and *κ*_*f*_ on simulated PIPO-SHG data. Effect of changing *ρ*_*f*_ (a), effect of changing *κ*_*f*_ assuming the real and imaginary parts are of equal magnitude (b), effect of changing *κ*_*f*_ with real and imaginary parts of different magnitude (c). Initial parameters are a fibril diameter of 280 nm, *ρ*_*f*_ = 1.90, and *κ*_*f*_ = 0.55(1 + i).

Numerical simulations were performed for collagen fibrils at varying positions on the focal plane of a PIPO-SHG microscope, and different values of *ρ* are obtained depending on the position of the fibril similar to the gradients observed in the experimental data. To better understand the origin of the gradient in *ρ* values across the fibrils, the effects of varying *ρ*_*f*_ and *κ*_*f*_ on the simulated gradient was investigated. It is found that increasing *ρ*_*f*_ results in an increase of the value of *ρ* at any given point in the focal volume, however there is little change to the gradient’s steepness between values [Figure 6(a)]. Increasing the magnitude of *κ*_*f*_ while keeping its real and imaginary parts equal is found to result in a steeper gradient with the *ρ* values becoming lower on one side of the fibril and higher on the other, but results in little change to the value of *ρ* at the center of the focal volume [Figure 6(b)]. This indicates that *ρ* values measured at the center of the fibril are the best indication of *ρ*_*f*_. When the sign of the imaginary component of *κ*_*f*_ is changed it results in a reversal of the gradient direction, and when a purely real value of *κ*_*f*_ is used the gradient disappears [Figure 6(c)]. This shows that the gradient is generated by the imaginary component of $\chi_{\mathrm{XY Z}}^{\left(2\right)}$ . The $\chi_{\mathrm{ZY Y}}^{\left(2\right)}$ term is considered to be real valued [15] (equal to $\chi_{\mathrm{ZXX}}^{\left(2\right)}$ in the case of C_6_ symmetry), therefore, it is not responsible for the gradient. A complex value of *κ*_*f*_ is required because the longitudinally polarized component of the focal field is out of phase from the main transverse component (see above) and therefore, a phase difference between *ρ*_*f*_ and *κ*_*f*_ is required to generate the gradient.

### Comparison of numerical simulations to PIPO-SHG results

**Figure 7:**
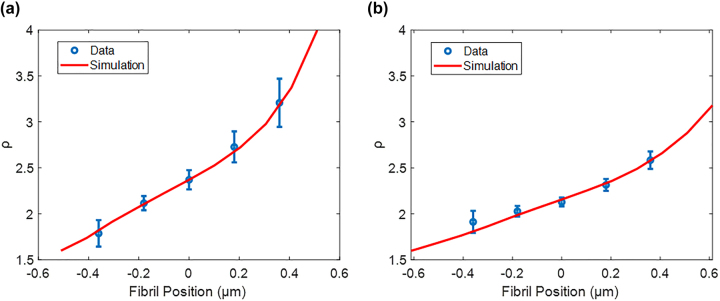
Typical result of fitting simulation to experimental data for two fibrils. The fibril in (a) has a diameter of 223 nm with manually fitted ratios of *ρ*_*f*_ = 2.10 ± 0.05 and *κ*_*f*_ = (0.8 ± 0.05) (1 + i) while the fibril in (b) has a diameter of 345 nm with *ρ*_*f*_ = 1.95 ± 0.05 and *κ*_*f*_ = (0.6 ± 0.05) (1 + i).

In order to validate the simulation results, profiles of *ρ* values across the fibrils were extracted for each of the 5 fibrils investigated. These fibrils were then imaged using AFM to determine their diameter, therefore making *ρ*_*f*_ and *κ*_*f*_ the only unknown simulation parameters, as well as to ensure that they were all single fibrils. Simulations are subsequently performed to manually fit the experimental *ρ* profiles data, while adjusting the values of *ρ*_*f*_ and the imaginary part of *κ*_*f*_ ( $\kappa_{f}^{\prime })$ in increments of 0.05. The results from each fibril are summarized in Table 1, and typical fits of the simulations to the experimental data are shown in Figure 7.

**Table 1:** Results of fitting simulations to experimental data.

**Diameter (nm) ± 3**	***ρ*_*f*_ ± 0.05**	${\kappa^{\prime }}_{f}$ **± 0.05**
223	2.10	0.80
246	1.75	0.45
280	1.70	0.35
329	1.90	0.50
345	1.95	0.60

## 4 Discussion

PSHG measurements of individual collagen fibrils show a gradient is consistently observed in measured *ρ* values transverse to the axis in individual fibrils. This gradient was initially unexpected since the focal spot of the PSHG microscope is much larger than the diameter of a typical collagen fibril. Because of this, each pixel across a PIPO-SHG image of a single fibril contains contributions from the whole fibril centered at different points on the focal plane. The gradient therefore is unlikely to be the result of variations in structure across the fibril and is more likely to arise as a result of non-uniformity within the focal electric field. This is further supported by experimental results which show a reduced gradient when the fibril is imaged using a low NA microscope objective compared to a high NA objective.

The numerical simulations of the focal volume for a high NA objective show a significant longitudinally polarized component that results in a high (>30 %) degree of circular polarization near the lateral edge of the focal volume, with opposite circular polarizations on each side of the focal volume, similar to what has been shown previously [42–44]. For individual collagen fibrils near the lateral edge of the focal volume, this circular polarization interacts with the complex valued $\chi_{\mathrm{XY Z}}^{\left(2\right)}$ component of the second order electric susceptibility, resulting in additional contributions to the measured SHG intensity that are not taken into account in Equation (1). These additional contributions are the likely source of the gradient in *ρ* values which we measure experimentally.

To our knowledge no previous work has reported an experimentally measured gradient in *ρ* across collagen fibrils. Numerical simulations have been used to investigate the effect of high NA focusing on PSHG measurements of collagen, however most of these models failed to consider the chirality of collagen and set *κ*_*f*_ to zero [40, 45, 46]. This resulted in an underestimation of the impact of high NA induced longitudinal polarization on SHG from collagen. Only numerical simulations which take into account the chirality of the collagen fibril have been successful in predicting the gradient [47]. Previous experimental work on PSHG of individual collagen fibrils either did not use sufficient magnification to observe this effect [30], or only report mean values of *ρ* rather than whole images [31]. Other studies using PSHG to investigate collagen structure have focused on collagen within tissues such as tendons, skin, cornea, cartilage and bone [14, 16], [17], [18], [19], [20], [21], [22]. In such collagen rich tissues, there are a large number of collagen fibrils within a single focal volume, and therefore there will typically be a fibril near the center of the focal spot which will produce a dominant SHG signal as compared to fibrils near the edge, and therefore, gradients are not typically observed in collagenous tissues.

By manually comparing numerical simulations of the collagen SHG to experimental data estimated values of *ρ*_*f*_ and $\kappa_{f}^{\prime }$ were obtained for each of the measured fibrils which are summarized in Table 1. This produced a range of values with *ρ*_*f*_ varying from 1.70 to 2.10, and $\kappa_{f}^{\prime }$ varying from 0.35 to 0.80. Note that the gradient arises from the imaginary part of *κ*_*f*_ (Figure 6c) so the real part is not constrained by our measurement. A likely explanation for this variation is that slight differences in hydration between fibrils led to changes in *ρ*_*f*_ and $\kappa_{f}^{\prime }$ (see Figure S1 in the supplementary material for a comparison of dried fibrils versus fibrils in water). Because of these variations we suggest that it may be more accurate to perform future measurements on fibrils in a humidity-controlled chamber.

Another possible explanation for this variation is that there are contributions from small amounts of other collagen types within the predominantly type I fibrils. Collagen type III is known to play a significant role in the formation of type I collagen fibrils, with the diameter and physical properties of the fibril depending on the presence of collagen type III during fibril formation. This likely results in a small percentage of type III tropocollagen molecules being embedded in the predominantly type I fibrils [48, 49]. This could have an impact on the observed PIPO-SHG data as it is known that collagen type III produces less SHG signal and gives different *ρ* values compared to type I [50–53]. Type V collagen is also known to be present in type I collagen fibrils. Type V collagen is thought to make up the core of the fibril and serve as a nucleation site for fibril assembly [54, 55]. Type V collagen does not produce significant SHG signal on its own [56] however, in combination with other collagen types it could alter the structural parameters similar to hydrogels resulting from mixed collagen I and V [57], and could therefore have an impact on PIPO-SHG measurements of collagen fibrils as well. The presence of different collagen types within a single fibril creates heterogeneity in the fibrils’ structure which was not modeled here. Better modeling of the distribution of collagen types within a single fibril, as well as better control over the fibril’s water content is expected to lead to lower uncertainty in estimating the values of *ρ*_*f*_ and $\kappa_{f}^{\prime }$ for a tendon fibril. Slight movement of the sample during imaging may also affect the values obtained however, simulations of a moving sample show that the change in gradients compared to a stationary sample are quite small (see Fig. S2 in supplementary material).

The mean values of *ρ*_*f*_ and $\kappa_{f}^{\prime }$ found here for 5 fibrils were *ρ*_*f*_ = 1.9 ± 0.2 and $\kappa_{f}^{\prime }$ = 0.5 ± 0.2. These values are in agreement with the previously reported values of *ρ*_*f*_ = 1.73 ± 0.01 for longitudinally cut pig tendon, and $\kappa_{f}^{\prime }$ = 0.34 ± 0.05 for transverse cut pig tendon [16]. However, we find that there are major changes in measured *ρ* values between fibrils in different hydration states, so without knowing the hydration states of the different samples it is difficult to make an accurate comparison (see Supplementary material Figure S1).

Comparing experimental data with numerical simulations it is possible to estimate the value of $\kappa_{f}^{\prime }$ for collagen fibrils, a quantity which is related to the chirality of the structure [58]. Previously, PSHG has been used for measurements of the chirality of collagen fibrils in tissues and thin films [13, 14, 16, 34, 59]. However, this typically requires that fibrils be tilted off the image plane (*α* > 0°) in order to fulfill Equation (2b), which is difficult to achieve for nanoscale structures such as a single collagen fibril, and in tissues where the presence of multiple fibrils with different orientations and diameters within the focal volume likely leads to inaccurate results.

It is interesting that the simulations show that a complex valued *κ*_*f*_ is required to generate the gradients in *ρ* which we observe experimentally. This is surprising since it is expected that all elements of *χ* ^(2)^ are real valued except near resonance [37]. A previous measurement of collagen SHG at various excitation wavelengths found no resonance enhancement of SHG signal near our excitation wavelength [60]. It has also been found that complex values of *κ*_*f*_ are required to produce circular dichroism in SHG intensity [16] which is a commonly reported parameter in collagen SHG literature [13, 20, 34, 59], and has been suggested as a potential parameter for differentiating normal and cancerous collagenous tissues [25, 61]. It has been suggested that this imaginary contribution to *κ*_*f*_ is the result of magnetic dipolar or electric quadrupolar contributions to SHG intensity [13, 59]. While the origins of electric dipole contributions to SHG have been well characterized [15, 62, 63], the structural origins of magnetic dipole and quadrupole contributions remains unknown.

The observation that the majority of fibrils have a gradient with the same direction, i.e. the same sign of $\kappa_{f}^{\prime }$ , is strong evidence that there are other contributions beyond the electric dipole approximation. Under the electric dipole approximation, we expect the sign of $\kappa_{f}^{\prime }$ to be related to the fibril’s polarity, and we therefore expect to measure an equal number of fibrils with each sign. This means that the apparent imaginary values of *κ*_*f*_ are likely the result of chiral magnetic or electric quadrupole contributions to SHG which have been shown not to change sign with fibril polarity [59]. Thus, the sign of $\kappa_{f}^{\prime }$ is likely the result of the fibril structure and not its orientation. However, while the structural origins of electric dipole contributions to collagen SHG have been well characterized [15, 62], the origins of magnetic dipole contributions have not. Therefore, the gradient analysis technique presented here provides a powerful new technique for investigating the structural origins of SHG in collagen.

The reported approach provides measurements of chirality for individual collagen fibrils using a standard microscope utilizing PIPO-SHG and may be extended to the characterization of other chiral nanostructures such as nano-disks [64] and nano-spirals [65]. Currently, chirality measurements of nanostructures using PSHG require samples to be held at an angle relative to the imaging plane [66, 67] and hence researchers cannot use standard microscopes since high NA objectives typically have low working distances limiting maximum tilt angles. Instead, chirality PSHG measurements on nanowires have been performed in bulk where either a larger laser beam is used to image a surface with many nanowires deposited on to it or specialized systems are used which sacrifice optical resolution by using high tilt angles to observe chirality. Analysis from these specialized systems is complicated by requiring a correction for Fresnel reflections which result in non-uniform transmission and reflection of the different polarization components. Therefore, the technique presented in this paper can be easily implemented to characterize parameters related to the structure and chirality of individual biological and synthetic nanostructures in a standard PSHG microscope.

## Supplementary Material

Supplementary Material Details
