# Supplementary material for: High numerical aperture imaging allows chirality measurement in individual collagen fibrils using polarization second harmonic generation microscopy
Source: Nanophotonics. 2023 Apr 14;12(11):2061–71. doi: 10.1515/nanoph-2023-0177 (PMC10193268; doi:10.1515/nanoph-2023-0177)
Supplement: Supplementary file 1 — Supplementary Material Details [file j_nanoph-2023-0177_suppl_001.docx]

High numerical aperture imaging allows chirality measurement in individual collagen fibrils using polarization second harmonic generation microscopy

MacAulay Harvey,^1^ Richard Cisek,^1^ Mehdi Alizadeh,^2,3,4^ Virginijus Barzda,^2,3,4^ Laurent Kreplak,^5,*^ and Danielle Tokarz^1,*^

^1^Department of Chemistry, Saint Mary’s University, 923 Robie Street, Halifax, Nova Scotia, B3H 3C3 Canada

^2^Department of Chemical and Physical Sciences, University of Toronto Mississauga, Mississauga, Ontario, L5L 1C6, Canada

^3^Department of Physics, University of Toronto, 60 St. George St, Toronto, Ontario, M5S 1A7, Canada

^4^Laser Research Center, Faculty of Physics, Vilnius University, Sauletekio Av. 9, LT-10222 Vilnius, Lithuania

^5^Department of Physics and Atmospheric Science and School of Biomedical Engineering, Dalhousie University, Halifax, Nova Scotia, B3H 4J5, Canada

**1. Supplementary Information**

*1.1. Effect of Hydration on Collagen fibrils*

To investigate the effect of water on the $\rho$ parameter measured for collagen, three fibrils were imaged using PIPO-SHG after being isolated and left to dry overnight as described in the main text. These fibrils were then rehydrated with a drop of reverse osmosis water and a coverslip with a parafilm spacer was placed on top to prevent evaporation of water during imaging. The same three fibrils were then imaged again in the hydrated state.

A typical result is shown in Fig. S1, we note that the wet fibrils produce a much lower SHG signal than dried fibrils and that the difference is too great to be explained by reflections from the second coverslip (compare the intensity images in Fig. S1 (a) and (b) and note that 1.6× more laser power was used for the wet fibril). Significant differences are found in the measured $\rho$ values between the dried and wet fibrils [compare Fig. S1 (c) and (d)]. By fitting a Gaussian function to the histograms of $\rho$ for all three fibrils [Fig. S1 (e)] we obtain the mean values of $\rho$ = 2.00 ± 0.01 for dried and $\rho$ = 1.43 ± 0.01 for wet fibrils. This shows that changes in hydration can alter the nonlinear optical properties of collagen, as was demonstrated by Stoller et.al [1]. This also explains the variation in $\rho$ values reported in the literature for collagen, with values in the range of 1.3 - 1.7 being typically reported for tendon collagen [2–10], but values in the range of 2.0-2.3 have been reported for collagen type I in diseased tissue samples, which have been dehydrated prior to imaging [11–14].

The $\rho$ value can be related to the pitch angle of the collagen triple helix ($p_{TH}$, the angle of the peptide backbone with respect to the molecular axis in the triple helical collagen molecule), as well as parameters which can be measured using X-ray diffraction by the following equations [15],

$\tan p_{TH}=\frac{2\pi R}{P} \frac{\rho}{2+\rho}=\cos^{2} p_{TH}$ (1)

where $R$ and $P$ are the radius and pitch of the collagen triple helix, respectively. Plugging in our measured $\rho$ values into equation (1) gives $p_{TH}$ values of 45.00 ± 0.07° for fibrils in the dried state and 49.78 ± 0.10° for fibrils in the hydrated state. X-ray diffraction studies of the collagen triple helix have found values of $R$ = 1.5 Å and $P$ = 9.5 Å for dry collagen which increase by 20% and 1.75%, respectively, when the molecule is hydrated [16,17]. This leads to expected values of $p_{TH}$ of 44.8° for dried and 49.4° for hydrated collagen triple helices in excellent agreement with our experimental data.


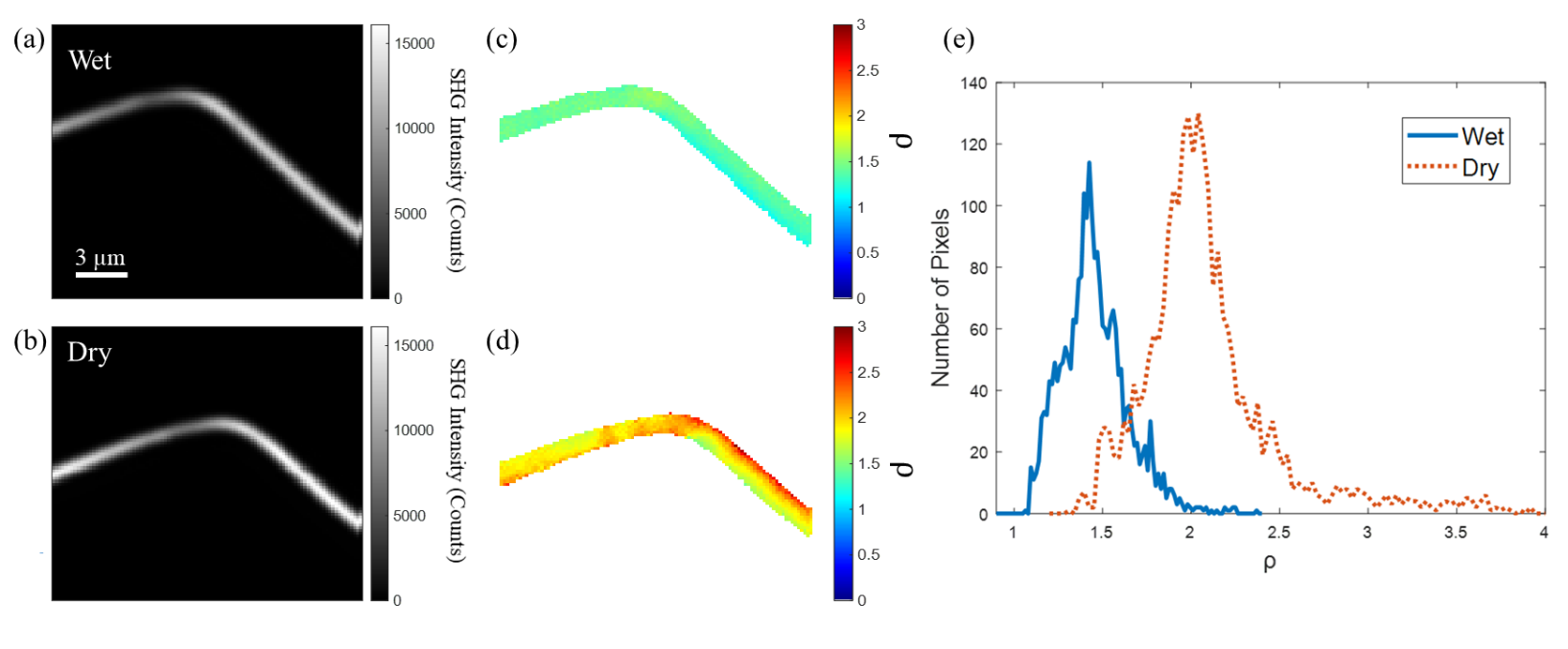


Fig. S1: The effect of hydration on PIPO-SHG of collagen fibrils. SHG intensity data for a typical wet (a) and dried (b) collagen fibril. Approximately 1.6 times more laser power was used for imaging the wet fibrils. Fitted $\rho$ values for the wet (c) and dry (d) fibril, and an occurrence histogram showing combined $\rho$ values for the same three fibrils (e).

*1.2. Effect of Sample Movement on PIPO-SHG Measurements*

The effect of sample movement on PIPO-SHG measurements of collagen fibrils was investigated for all five fibrils reported in the main text. To quantify sample movement the first eight images of PIPO-SHG data were summed, and a line profile 6 pixels wide was taken transverse to the fibril, a Gaussian function was fitted to determine the centroid position of the fibril. This was repeated for the last eight PIPO-SHG images to determine how much the sample moved during the PIPO-SHG scan. The minimum movement found was 0.22 pixels, the average was 0.50 pixels and the maximum was 0.72 pixels, recall that our microscope described in the main text has a pixel size of 180 nm.

To determine the effect that movement has on measured gradients across the fibril we simulate PIPO-SHG from fibrils where the center position of the fibril is adjusted after every eight input polarizations to simulate movement. We find that movement has a minor effect on the $\rho$ gradient, particularly near the edge of the fibril, but even the simulation of our maximum measured sample movement was within our typical uncertainty of the no movement case for four out of the five pixel locations which we measured (Fig. S2).

Fig. S2: Effect of sample movement on measured gradients. Plot showing simulated gradients in $\rho$, and mean uncertainty in experimental data at various points for a sample which is stationary (solid blue line), one which moves by 0.5 pixels, the mean movement measured here during the PIPO-SHG scan (orange dashed line), and one which moves 0.7 pixels, the maximum measured here during the PIPO-SHG scan.

References

[1] P. Stoller, K. M. Reiser, P. M. Celliers, and A. M. Rubenchik, "Polarization-modulated second harmonic generation in collagen," Biophys J **82**, (2002).

[2] S. Roth and I. Freund, "Second harmonic generation in collagen," J Chem Phys **70**, 1637–1643 (1979).

[3] I. Gusachenko, V. Tran, Y. G. Houssen, J. M. Allain, and M. C. Schanne-Klein, "Polarization-resolved second-harmonic generation in tendon upon mechanical stretching," Biophys J (2012).

[4] A. S. Quigley, S. Bancelin, D. Deska-Gauthier, F. Légaré, L. Kreplak, and S. P. Veres, "In tendons, differing physiological requirements lead to functionally distinct nanostructures," Sci Rep (2018).

[5] A. Golaraei, L. Kontenis, K. Mirsanaye, S. Krouglov, M. K. Akens, B. C. Wilson, and V. Barzda, "Complex Susceptibilities and Chiroptical Effects of Collagen Measured with Polarimetric Second-Harmonic Generation Microscopy," Sci Rep **9**, (2019).

[6] A. Golaraei, K. Mirsanaye, Y. Ro, S. Krouglov, M. K. Akens, B. C. Wilson, and V. Barzda, "Collagen chirality and three-dimensional orientation studied with polarimetric second-harmonic generation microscopy," J Biophotonics **12**, (2019).

[7] A. E. Tuer, M. K. Akens, S. Krouglov, D. Sandkuijl, B. C. Wilson, C. M. Whyne, and V. Barzda, "Hierarchical model of fibrillar collagen organization for interpreting the second-order susceptibility tensors in biological tissue," Biophys J **103**, (2012).

[8] D. Rouède, E. Schaub, J.-J. Bellanger, F. Ezan, and F. Tiaho, "Wavy nature of collagen fibrils deduced from the dispersion of their second-order nonlinear optical anisotropy parameters ρ," Opt Express **28**, (2020).

[9] F. Tiaho, G. Recher, and D. Rouède, "Estimation of helical angles of myosin and collagen by second harmonic generation imaging microscopy," Opt Express **15**, (2007).

[10] D. Rouède, E. Schaub, J. J. Bellanger, F. Ezan, J. C. Scimeca, G. Baffet, and F. Tiaho, "Determination of extracellular matrix collagen fibril architectures and pathological remodeling by polarization dependent second harmonic microscopy," Sci Rep **7**, (2017).

[11] A. Golaraei, L. B. Mostaço-Guidolin, V. Raja, R. Navab, T. Wang, S. Sakashita, K. Yasufuku, M.-S. Tsao, B. C. Wilson, and V. Barzda, "Polarimetric second-harmonic generation microscopy of the hierarchical structure of collagen in stage I-III non-small cell lung carcinoma," Biomed Opt Express **11**, (2020).

[12] D. Tokarz, R. Cisek, A. Joseph, A. Golaraei, K. Mirsanaye, S. Krouglov, S. L. Asa, B. C. Wilson, and V. Barzda, "Characterization of pancreatic cancer tissue using multiphoton excitation fluorescence and polarization-sensitive harmonic generation microscopy," Front Oncol (2019).

[13] D. Tokarz, R. Cisek, A. Joseph, S. L. Asa, B. C. Wilson, and V. Barzda, "Characterization of pathological thyroid tissue using polarization-sensitive second harmonic generation microscopy," Laboratory Investigation **100**, (2020).

[14] K. Mirsanaye, L. Uribe Castaño, Y. Kamaliddin, A. Golaraei, R. Augulis, L. Kontenis, S. J. Done, E. Žurauskas, V. Stambolic, B. C. Wilson, and V. Barzda, "Machine learning-enabled cancer diagnostics with widefield polarimetric second-harmonic generation microscopy," Sci Rep **12**, (2022).

[15] A. Leray, L. Leroy, Y. le Grand, C. Odin, A. Renault, V. Vié, D. Rouède, T. Mallegol, O. Mongin, M. H. V. Werts, and M. Blanchard-Desce, "Organization and orientation of amphiphilic push-pull chromophores deposited in Langmuir-Blodgett monolayers studied by second harmonic generation and atomic force microscopy," Langmuir **20**, (2004).

[16] K. Beck and B. Brodsky, "Supercoiled protein motifs: The collagen triple-helix and the α- helical coiled coil," J Struct Biol **122**, (1998).

[17] A. Masic, L. Bertinetti, R. Schuetz, S. W. Chang, T. H. Metzger, M. J. Buehler, and P. Fratzl, "Osmotic pressure induced tensile forces in tendon collagen," Nat Commun **6**, (2015).
